# A Broad Approach to Abrupt Boundaries: Looking Beyond the Boundary at Soil Attributes within and Across Tropical Vegetation Types

**DOI:** 10.1371/journal.pone.0060789

**Published:** 2013-04-10

**Authors:** Laura Warman, Matt G. Bradford, Angela T. Moles

**Affiliations:** 1 Evolution and Ecology Research Centre, School of Biological, Earth and Environmental Sciences, The University of New South Wales, Sydney, New South Wales, Australia; 2 Institute of Pacific Islands Forestry, United States Department of Agriculture Forest Service, Hilo, Hawaii, United States of America; 3 Ecosystem Sciences, Commonwealth Scientific and Industrial Research Organisation, Atherton, Queensland, Australia; University of Konstanz, Germany

## Abstract

Most research on boundaries between vegetation types emphasizes the contrasts and similarities between conditions on either side of a boundary, but does not compare boundary to non-boundary vegetation. That is, most previous studies lack suitable controls, and may therefore overlook underlying aspects of landscape variability at a regional scale and underestimate the effects that the vegetation itself has on the soil. We compared 25 soil chemistry variables in rainforest, sclerophyll vegetation and across rainforest-sclerophyll boundaries in north-eastern Queensland, Australia. Like previous studies, we did find some contrasts in soil chemistry across vegetation boundaries. However we did not find greater variation in chemical parameters across boundary transects than in transects set in either rainforest or woodland. We also found that soil on both sides of the boundary is more similar to “rainforest soil” than to “woodland soil”. Transects in wet sclerophyll forests with increasing degrees of rainforest invasion showed that as rainforest invades wet sclerophyll forest, the soil beneath wet sclerophyll forest becomes increasingly similar to rainforest soil. Our results have implications for understanding regional vegetation dynamics. Considering soil-vegetation feedbacks and the differences between soil at boundaries and in non-boundary sites may hold clues to some of the processes that occur across and between vegetation types in a wide range of ecosystems. Finally, we suggest that including appropriate controls should become standard practice for studies of vegetation boundaries and edge effects worldwide.

## Introduction

Boundaries between different vegetation types occur throughout the world in a variety of environments and range in scale from localized communities to interfaces that span hemispheres (such as the 13,000 km of boreal boundary between tundra and taiga) [1], [2]. Abrupt boundaries between vegetation types are an especially dramatic landscape component where two distinct vegetation forms (e.g. tropical rain forest and fire-prone savannas) abut against each other rather than being separated by a wide ecotone. These abrupt boundaries have long intrigued researchers and the role of soil in delimiting vegetation and maintaining these boundaries continues to be debated (see [3]–[5]).

Most studies approach boundaries as the limits of two vegetation types and the conditions that each type requires. Thus, studies tend to focus on the differences found directly across individual boundaries; many are carried out on single sites (often using single transects), and many focus solely on one of the vegetation types involved [2], [3], [6]–[8]. These approaches have undoubtedly increased our understanding of the factors involved in determining boundary locations and dynamics. However, individual boundary dynamics can be highly idiosyncratic [9]–[11] and by focusing on boundaries alone we may overlook or underestimate underlying aspects of landscape heterogeneity and long-term history at a regional scale. By treating boundaries as static vegetation limits we risk overlooking the effects of the vegetation itself on the parameters being measured, the effect of ecosystem-level processes (such as succession and decomposition) occurring in the boundary zone, and the dynamic nature of many boundaries (e.g. [1], [11]). In this study we approach abrupt boundaries between rain forest and sclerophyll woodland vegetation at a regional scale in the Australian Wet Tropics, specifically trying to incorporate local diversity of both soils and vegetation types, as well as acknowledging soil-vegetation feedbacks. We address an existing knowledge gap by comparing soil chemistry under boundaries to ‘controls’ in non-boundary vegetation (rain forest and sclerophyll woodland far from boundaries) and investigate the changes that occur in soil following the shift from wet sclerophyll forest to rain forest.

Abrupt boundaries between tropical rain forest and open, fire-prone vegetation can be found throughout the world, notably in South America [8], [12], [13], equatorial Africa [14], [15], India [16], New Caledonia [17], and northern Australia [18]–[20]. Fire has a critical role in boundary stability and maintenance in all of these systems, but it is generally accepted that no single factor is solely responsible for these abrupt boundaries [16], [21], [22]. Rather, rainforest and fire-prone vegetation are increasingly regarded as alternative stable states (ASS) which are maintained by a combination of suitable environmental factors together with feedbacks created by the vegetation itself [13], [23]–[25].

Studies around the world have shown that both boundaries and vegetation types have moved back and forth across the landscape through time, and that these shifts can occur relatively quickly (e.g. [1], [11]). In a system with ASS, we would expect to find both sharp boundaries, and rapid shifts between vegetation types [23], [25]. In North Queensland rainforest margins expand, on average, more than half a metre per year [11], and some boundaries have been shown to shift horizontally more than a metre per year [11], [21]. This means that studies which have compared soil directly across boundaries with the assumption of comparing two types of soil (i.e. “woodland soil” abutting against “rain forest soil”) may actually be comparing two types of “boundary soil”. It is impossible to know whether or not this is the case without using regional controls under non-boundary vegetation. However, no previous studies have used this type of controls. Rather, studies have compared adjacent contrasting vegetation (e.g. [2], [19]), looked at variations in soil within a single type of vegetation (e.g. [26]), compared fine-scale differences in either vegetation near the boundary (e.g. [8]) or compared soil under vegetation types at a regional scale without comparing boundaries (e.g. [27]). The first aim of our study is to compare soil chemistry across boundaries with the corresponding vegetation type from independent non-boundary sites to ascertain whether the soil on either side of the boundary is actually representative of “sclerophyll woodland soil” and “rain forest soil”, or more closely resembles “boundary zone soil”.

Globally, abrupt boundaries between rain forest and open vegetation are characterised by strong contrasts in canopy cover, tree density, plant biomass and species composition as well as soil moisture and other microsite conditions (including light penetration, wind speed, temperature and leaf litter accumulation) [13], [17], [22], [28]–[30]. In contrast to these clear and consistent differences, patterns in soil characteristics (other than soil moisture) remain equivocal even at local scales. Some studies report strong contrasts in soil chemistry across boundaries [13], while others report weaker and/or contradictory trends even in factors which would be expected to be relatively consistent (such as pH levels) [6], [12], [31]. While these inconsistencies may reflect inherent differences between boundaries, they may also be a product of sampling artefacts caused by not taking into account variability at non-boundary sites, nor processes (such as feedbacks) occurring at the boundary zone. The second aim of our study is to use controls in non-boundary vegetation, to test whether contrasts across boundaries (measured as within-boundary transect variance) are actually higher than would be expected given the regional heterogeneity of conditions that either vegetation type is found in (measured as within-single vegetation type transect variances).

There is increasing interest in and discussion of vegetation-soil feedbacks and of how vegetation itself affects soil parameters [3], [13], [16], [32]. The effects of these feedbacks are especially relevant in systems where the vegetation is thought to function as ASS. Numerous studies have shown the effects on soil chemistry of removing rain forest vegetation or of re-vegetation following clearing [15], [33]–[35]. Less emphasis has been placed on tracking changes in the soil during natural shifts from open to closed forest vegetation. Numerous studies looking at charcoal, soil carbon content, isotope ratios and radiocarbon dating have shown that both forests and grasslands can leave “footprints” when they replace each other on the landscape [9], [15], [20], [36], but the effects of vegetation change on other aspects of soil chemistry remain unclear. The third aim of our study is to quantify the effects of the vegetation itself on the soil. Specifically, to quantify the effects of rain forest replacing wet sclerophyll vegetation in a regional context. If the differences in soil under rain forest and woodland are, at least partly, due to an effect of the vegetation types themselves, then as rain forest expands into wet sclerophyll forest, the soil under the latter should progressively change to resemble the soil under rain forest.

In summary, the hypotheses we test are:

1. The soil chemistry on either side of the boundary corresponds more closely to “boundary zone” soil, rather than either rain forest or woodland soil;
2. The contrasts in soil chemistry across any rain forest-woodland boundary transect are higher than the variability found within any transect set within a single vegetation type,
3. There is a measurable effect on the soil from natural changes in the vegetation over time, and, as rain forest replaces wet sclerophyll forest, the soil under the former will increasingly resemble the latter.

## Methods

### Ethics Statement

Permission to sample vegetation and soil was obtained from the Queensland Government Environmental Protection Agency (permit numbers WITK05321808, WISP05321908).

### Study Region

**Figure 1:**
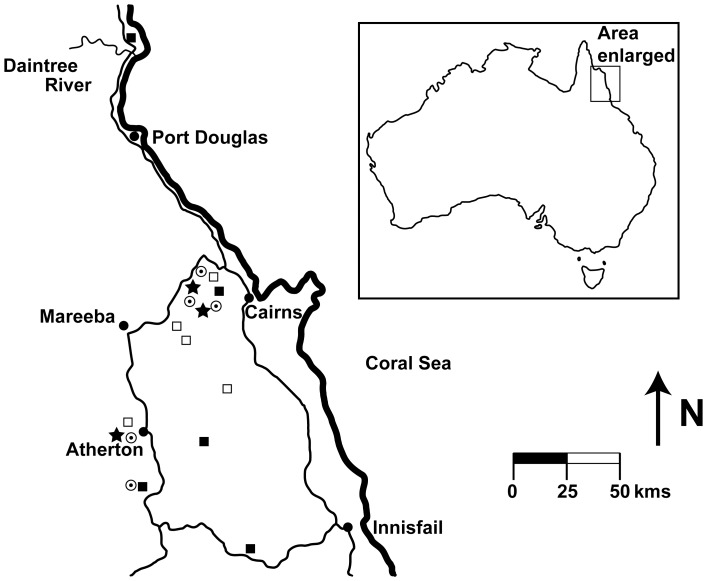
Map of the 18 transect locations sampled in 2009. Map of the 18 transect locations sampled in 2009. Filled squares represent RF sites, open squares represent SF sites, open circles represent boundary sites and stars represent WSF sites. Major roads and towns (filled circles) are also indicated.

The study took place in the Wet Tropics bioregion of north-eastern Queensland, Australia (Figure 1). The vegetation of the Australian Wet Tropics is characterised by a mosaic of rain forests (RF) and more open, fire-prone sclerophyll vegetation (SF; Table 1), often with abrupt boundaries between them [18], [21], [28], [37], [38](Figure S1). The region as a whole is also characterised by high variability in soils, topography, rainfall and climate [39], [40]. Maximum rainfall occurs over summer, while the dry season extends between May and October, but the region is not as strongly seasonal as the Wet Dry Tropic region found further north and in the Northern Territory [3], [39].

**Table 1:** Vegetation types considered in this study.

Vegetation type	Description	Global analogues
Rain forest (RF)	Closed canopy forests with heavily shaded understories. Canopyheight approx. 30–35 m. Common life forms include palmsand ferns and vines and creepers. [3], [39] (Figure S3)	The RF of the Australian Wet Tropics are analogous to lowland rainforest, lowland wet forest, and pre-montane rainforest elsewhere.
Sclerophyll vegetation(SF)	Pyrophytic vegetation types ranging from grassland and savannato woodland and dry forest dominated by eucalypts. Much moreopen than closed canopy rainforest, with a higher proportionof grass in the understory [3], [57] (Figure S2)	Some authors [11], [30] use ‘savanna’ as shorthand for this vegetation type in the Australian Wet Tropics. However, for the majority of transects included in this study (Table S1), ‘woodland’ or ‘dry forest’ is a more appropriate equivalent.
Boundary vegetation(BnRFand BnSF)	We define boundary vegetation in our study as the vegetationextending 30 m to each side of an abrupt boundary betweenRF and SF (Figure S1).	
Wet SclerophyllForest (WSF)	Tall open forest with a mesic understory, ranging from grassesand sedges to developing RF communities. WSF occurs inareas with relatively high rainfall [37]–[39] (Figure S4)	Has some functional similarities with the ‘cerradão’ forests of South America (sensu [55]), but canopy height is considerably taller, and WSF are mostly characterized by the presence of certain eucalypt species (such as *E. grandis*, *E. saligna* or *E. regnans*)

Another characteristic of the vegetation of the Australian Wet Tropics is the presence of a narrow band of tall open wet sclerophyll forests (WSF; Figure S4) that can form an ecotone between RF and SF vegetation. These forests contain a mixture of species and processes from both RF and SF vegetation (Table 1) [23]. Under the right conditions, and in the absence of fire, RF can rapidly expand into, and eventually replace, WSF vegetation (a process locally referred to as ‘rainforest invasion’) [41]. These forests offer an interesting opportunity to test ideas about vegetation-soil feedbacks.

### Site Selection

Sites were chosen to reflect and incorporate the broad regional diversity across and within vegetation types (Table 1, Table S1, Figures S1, S2, S3, and S4). Thus, sites range from coastal RF in Daintree National Park through to the Atherton and Herberton Tablelands as well as SF vegetation throughout the Lamb and Herberton ranges. Sites range in altitude from 280 to 847 masl (Figure 1 and Table S1), and regionally rainfall varies from around 900 mm year^−1^ in the Lamb Range to over 3000 mm year^−1^ (near Innisfail).

We chose relatively undisturbed sites, avoiding intersecting waterways, riparian gallery forests and areas extensively covered by invasive species. We aimed to sample relatively flat areas to avoid runoff or down-slope concentration of nutrients (but when necessary, transects were set across the slope face). Boundary sites were chosen on the basis of strong contrasts in canopy closure and species composition, particularly reflected in the density of grass cover. Two boundary transects (Surprise Creek and Herberton) were intersected by a trail which could ostensibly function as a firebreak. Non-boundary sites were located several kilometres away from the nearest boundary.

### Soil Collection and Analysis

Sampling was carried out during April of 2009, at start of the dry season. Eighteen transects were laid out; five each in RF, SF and across abrupt boundaries between these two vegetation types. Three additional transects were set up in WSF sites with varying degrees of invasion by RF (see below). All transects were 60 m long and pooled samples were collected at 12 m intervals along each transect, for a total of 107 pooled samples. Each pooled sample consisted of five smaller samples taken to a depth of 15 cm within one square metre of the transect line. Leaf litter and other detritus were brushed away prior to sampling. Parent material was assessed on site. The starting points for transects were randomly chosen and, in the case of boundary transects, three sampling points were placed on each side of the boundary.

Soil samples were dried at 70°C for at least 48 hours then passed through a sieve to remove roots, woody material and small rocks greater than approximately 15 mm in diameter. Dried samples were crushed using a TEMA mill (TEMA Machinery Limited, Northants, UK) at UNSW and then a suite of 25 general soil chemistry and fertility parameters was measured at the Environmental Analysis Laboratory at Southern Cross University (Lismore, NSW, Australia). Measured parameters included organic matter content, macronutrients (carbon, nitrogen, phosphorus, calcium, magnesium, potassium and sulphur), pH, conductivity, and some micronutrients (Table 2). Phosphorus tests included available P Bray I, potentially available P Bray II and total P Colwell.

**Table 2:** Parameter values and tests of significance.

	SF	Bn SF	Bn RF	RF	SF vs RF	BnSF vs BnRF	SF vs BnSF	RF vs BnRF	SF vs BnRF	RF vs BnSF
Organic matter	6.18±4.74	11.66±4.9	13.73±6.03	12.55±5.91	<0.001	n. s.	0.001	n. s.	<0.001	n. s.
pH (1∶5 water)	6.02±1.18	5.15±0.43	5.13±0.3	5.66±0.61	n. s.	n. s.	0.029	0.009	0.021	0.015
eC µS cm^−1^	86.26±28.53	110.46±28.23	147.93±36.01	191.80±76.37	<0.001	0.001	0.02	0.08	<0.001	0.001
CEC	6.90±3.11	8.56±3.01	9.95±4.02	20.25±18.29	n. s.	n. s.	n. s.	n. s.	n. s.	n. s.
C%	3.53±2.71	6.66±2.8	7.84±3.45	7.17±3.38	<0.001	n. s.	0.002	n. s.	<0.001	n. s.
N%	0.14±0.12	0.32±0.14	0.42±0.18	0.54±0.28	<0.001	0.005	<0.001	n. s.	<0.001	0.007
C:N	29.44±12.38	20.77±3.78	18.28±2.18	13.60±1.97	<0.001	0.001	0.005	<0.001	<0.001	<0.001
NO_3_ ppm	1.62±1.07	2.33±1.21	3.26±1.53	7.14±5.2	<0.001	0.019	n. s.	0.005	0.001	<0.001
NH_4_ ppm	9.90±7.07	20.12±10.98	28.96±12.36	43.32±9.02	<0.001	0.008	0.002	n. s.	<0.001	0.004
P (Bray I)	3.53±4.62	3.48±4.86	4.94±6.53	2.53±2.74	n. s.	n. s.	n. s.	n. s.	n. s.	n. s.
P (Bray II)	8.72±6.84	8.06±5.35	10.80±7.15	42.21±59.11	n. s.	n. s.	n. s.	n. s.	n. s.	n. s.
P (Colwell)	12.05±10.65	17.82±9.02	20.73±10.04	50.38±48.84	<0.001	n. s.	0.017	0.040	0.002	0.008
Ca kg ha^−1^	1566.18±1322.8	1167.46±922.68	1562.47±811	6080.80±6833	n. s.	n. s.	n. s.	n. s.	n. s.	n. s.
Mg kg ha^−1^	370.37±153.41	467.97±145.54	692.74±276	1157.48±984.2	<0.001	n. s.	0.037	n. s.	0.001	n. s.
Ca:Mg	2.63±1.92	1.49±0.93	1.42±0.63	2.30±1.63	n. s.	n. s.	n. s.	n. s.	n. s.	n. s.
K kg ha^−1^	898.51±402.62	652.26±374.43	703.9±370.36	745.901±335.2	n. s.	n. s.	n. s.	n. s.	n. s.	n. s.
Al kg ha^−1^	108.16±176.85	482.11±322.9	636.77±479.7	210.87±244.1	n. s.	n. s.	<0.001	<0.001	<0.001	<0.001
Mn ppm	20.19±14.47	14.83±12.4	46.92±41.06	75.03±53.31	0.011	<0.001	n. s.	n. s.	n. s.	0.005
Fe ppm	140.77±116.59	260.65±96.22	271.59±80.25	295.95±144.3	<0.001	n. s.	<0.001	n. s.	<0.001	n. s.
Cu ppm	0.52±0.26	0.77±0.6	1.31±1.26	2.07±0.97	n. s.	n. s.	n. s.	n. s.	n. s.	n. s.
B ppm	0.40±0.11	0.68±0.24	0.95±0.35	0.95±0.39	<0.001	0.003	<0.001	n. s.	<0.001	n. s.
Sulphate S ppm	12.77±13.43	29.32±10.19	38.95±12.36	41.32±9.02	<0.001	0.001	<0.001	0.42	<0.001	0
Na kg ha^−1^	114.88±37.49	97.15±21.26	112.3±49.41	129.08±92.1	n. s.	n. s.	n. s.	n. s.	n. s.	n. s.
Si ppm	86.57±34.87	135.71±58.02	163.95±68.36	165.09±84.66	<0.001	n. s.	0.01 1	n. s.	<0.001	n. s.
Zn ppm	1.39±1.1	1.26±0.73	1.69±1	4.46±5.13	n. s.	n. s.	n.s.	n. s.	n. s.	n. s.

Mean values (±Std Dev) for soil parameters measured in sclerophyll vegetation (SF), rain forest (RF), boundary sclerophyll vegetation (BnSF) and boundary rain forest vegetation (BnRF); as well as degrees of significance for comparisons (using a linear mixed effect model in R, to account for within-transect variation) between parameters at each of these vegetation types.

### Wet Sclerophyll Forest Chronosequence Data

As a proxy for a chronosequence, three transects were set up in WSF sites, each one with an increasing degree of invasion by RF. We chose sites where *Eucalyptus grandis* was present in the canopy because this species can survive under RF, but requires fire and relatively open conditions to establish and regenerate [38]. Hence, the presence of *E*. *grandis* as canopy species in a mature RF indicates a vegetation shift through time from open vegetation to closed forest [37].

The vegetation at the three WSF sites corresponds to WSF types I, II and III as described by [38], with *E. grandis* present and RF invasion increasing from type I to III. At the type I site (Herberton) a mature canopy of *E. grandis* grows over an understorey mixture of grasses, sedges and shrubs. Type II (at Clohesy River), represents the next progressive stage, with a stand of mature *E. grandis* and *E. grandis*/*Eucalyptus pellita* hybrids as emergents over a developing RF understorey. At the type III site (Smith’s track) a few relict emergent *E. grandis* survive in an established mature RF with a canopy around 35 m high. We could find no regenerating eucalypts at this site.

In addition to the data from these three transects, we also analysed a dataset collected in the dry season of 1995. This dataset includes measurements of phosphorus (%), carbon (%), nitrogen (%) and electro-conductivity (µS cm^−1^) for 235 soil samples from WSF types I, II and III collected from transects at Baldy Range, the Carbine Tablelands, Herberton Range, Kirrima Range, Koombooloomba, Lamb Range, Paluma Range, Wallaman Falls and the Windsor Tableland. The pooled 1995–2009 dataset thus includes 252 samples spanning nearly three degrees of latitude, approximately 350 km north to south and 60 km inland from the coast.

### Statistical Analysis

All soil parameter measurements, except calcium, magnesium and sodium, were log_10_ transformed prior to analysis.

We compared the values of soil parameters across boundaries and between boundary and non-boundary vegetation by comparing the regional data while taking into account the variation between transects. To do this we used a linear mixed effect model in R (lmer; [42]) with a random effect for site (i.e. transect) to compare all parameters in RF, SF, boundary-rain forest (BnRF) and boundary-sclerophyll vegetation (BnSF).

We calculated the variance for each parameter measured within each transect and then compared the variances found within each transect, for each parameter, across transect types (boundary, RF and SF) using a general linear model (GLM) with fixed effect for vegetation type in SPSS (v. 17.0). We used this approach rather than a comparison of values from the three BnSF and three BnRF sites on each transect because it allows us to make meaningful, consistent comparisons between boundary transects and control transects within vegetation types (RF transects and SF transects). This is especially important given the broad range of ecological and environmental variation comprised within our study region. Comparing the magnitude of the variance, allows us to test whether contrasts across boundaries are greater than what would be expected given the regional heterogeneity of both environmental conditions and vegetation types (i.e. differences among rainforests and sclerophyll vegetation types).

Linear mixed effect models in R were used again to compare the soil parameters in the samples from WSF with increasing levels of RF invasion where the model included a random effect for site. We repeated the tests using only samples that had either granite or rhyolite as parent material (all samples from basalt were in type I forest) to ascertain whether our results were influenced by parent material, but results were qualitatively unchanged in these tests.

## Results

### Soil Parameters Across Boundaries and Vegetation Types

**Figure 2:**
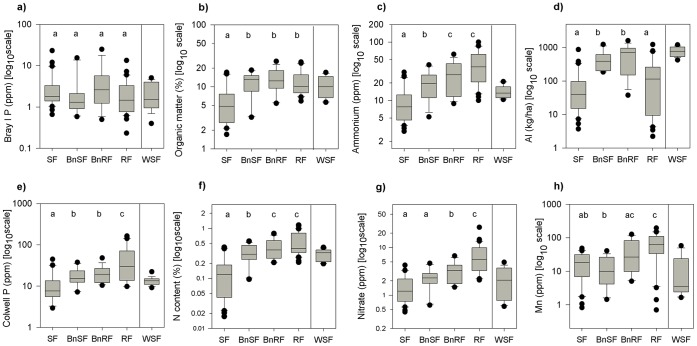
Soil parameters. Soil parameters measured in sclerophyll vegetation (SF), rain forest (RF), wet sclerophyll forest (WSF) and along transects that crossed boundaries between boundary sclerophyll vegetation (BnSF) and boundary rain forest vegetation (BnRF). Box plots mark the median and span from the 25^th^ to the 75^th^ percentiles, with error bars at the 10^th^ and 90^th^ percentiles. WSF values included as a comparison. a)-d) Show the four main patterns in our data: a) no difference in soil between vegetation types (36%); b) a difference between SF values and those from the other vegetation types (20%); c) no difference between RF and BnRF, but these are different to both SF and the BnSF (16%); d) a difference between boundary vegetation and non-boundary vegetation. a) And e) show different results for soil phosphorus, while c) and g) show different forms of nitrogen (f). Manganese in f) shows a pattern which could be strongly misinterpreted if only boundary values had been measured.

We found differences between RF and SF soils, as well as across boundaries, however the patterns across the boundaries were not always consistent with the differences between RF and SF soils. Nine parameters (36%) showed no significant difference between any of the vegetation types (Table 2, Figure 2). These included cation exchange capacity (CEC; a measure of soil fertility related to the capacity of the soil to retain cations), both Bray (I and II) phosphorus tests, calcium, Ca:Mg ratios, potassium, copper, sodium and zinc.

#### Differences between SF and RF

Fourteen out of 25 measured parameters (56%) differed significantly between the RF and SF transects (that is, the control transects in non-boundary vegetation; Table 2). Soil organic matter, electrical conductivity, carbon, nitrogen, iron, boron, silicon, magnesium and sulphate sulphur levels were, on average, two to three times higher in RF than in SF (C:N ratios were accordingly twice as high in SF than in RF). Phosphorus (Colwell) and manganese were four times larger in RF.

#### Differences between boundary and non-boundary vegetation

When comparing the boundary vegetation (BnRF and BnSF) to their corresponding controls (i.e. RF and SF; Table 2), we found that BnRF was generally more similar to RF (significant differences in 7 out of 25 parameters; 28%) than was BnSF to SF (significant differences in 14 out of 25 parameters; 56%). Nearly a quarter of our results showed that soil on both sides of the boundaries was more similar to RF soil than to SF soil (Figure 2), and we found no cases where soil parameters in RF and BnRF were significantly different to SF and BnSF. In other words, rather than find a pattern where soil showed no difference under corresponding boundary and control vegetations (e.g. RF and BnRF), but showed differences between both groups of soils (i.e. RF and BnRF vs SF and BnSF), we found a variety of idiosyncratic patterns where soil parameter values where most likely to be most similar to rainforest soil values. Values for soil organic matter, electrical conductivity, carbon, Colwell phosphorus, magnesium, iron, boron and silicon increased by less than double from SF to BnSF, while nitrogen, ammonium and sulphur sulphate more than doubled. C:N ratio and pH were both less than 1.4 times larger in SF than BnSF. When comparing parameters from RF to BnRF, electrical conductivity, pH and sulphate sulphur showed minimal increases (roughly 1.1 times greater in RF) with a similar decrease in C:N (1.3 times greater in BnRF) while nitrate and Colwell phosphorus increased by more than double in RF.

Boundary vegetation showed a significantly lower pH (*p* = 0.01) and significantly higher aluminium (*p*<0.001) than did SF and RF, which did not differ significantly from each other. Soils under SF displayed a wide range of pH values from highly alkaline (8.8 on Mt. Baldy) to highly acidic (3.8 at Surprise Creek), while RF soils showed a narrower and more acidic range (6.4 to 4.5). The largest parameter differences between boundary and non-boundary vegetation were found in levels of aluminium, which were 4.4 times higher in BnSF and 3 times higher in BnRF than in non-boundary controls.

#### Differences across vegetation boundaries

Eight out of 25 soil parameters (32%) varied significantly across boundaries, and the magnitude of these variations was smaller than those between SF and RF. Electrical conductivity, boron and silicon, plus nitrogen and related measures of ammonium and nitrate, were all roughly 1.4 times higher in BnRF than in BnSF, while C:N ratio was 1.1 times higher in BnSF than BnRF. Manganese showed the highest difference, being three times higher in BnRF than BnSF (Table 2). Neither organic matter, pH, carbon, magnesium nor phosphorus differed across boundaries (Figure 2), even though they differed between SF and RF.

Lastly, total nitrogen, ammonium and nitrate all increased from SF to RF but showed different contrasts between the four vegetation types (Figures 2c, 2f, 2g). Ammonium and total nitrogen were highest in RFs and BnRF, then dropped at the BnSF and again in SFs. Nitrate was the only parameter whose values showed no difference (*p* = 0.12) between SFs (1.6 ppm) and BnSF (2.3 ppm). These values were lower than those of BnRF (mean 3.3 ppm; *p*<0.02) and RF itself (7.1 ppm; *p*<0.001).

### Variance Across Transects

**Figure 3:**
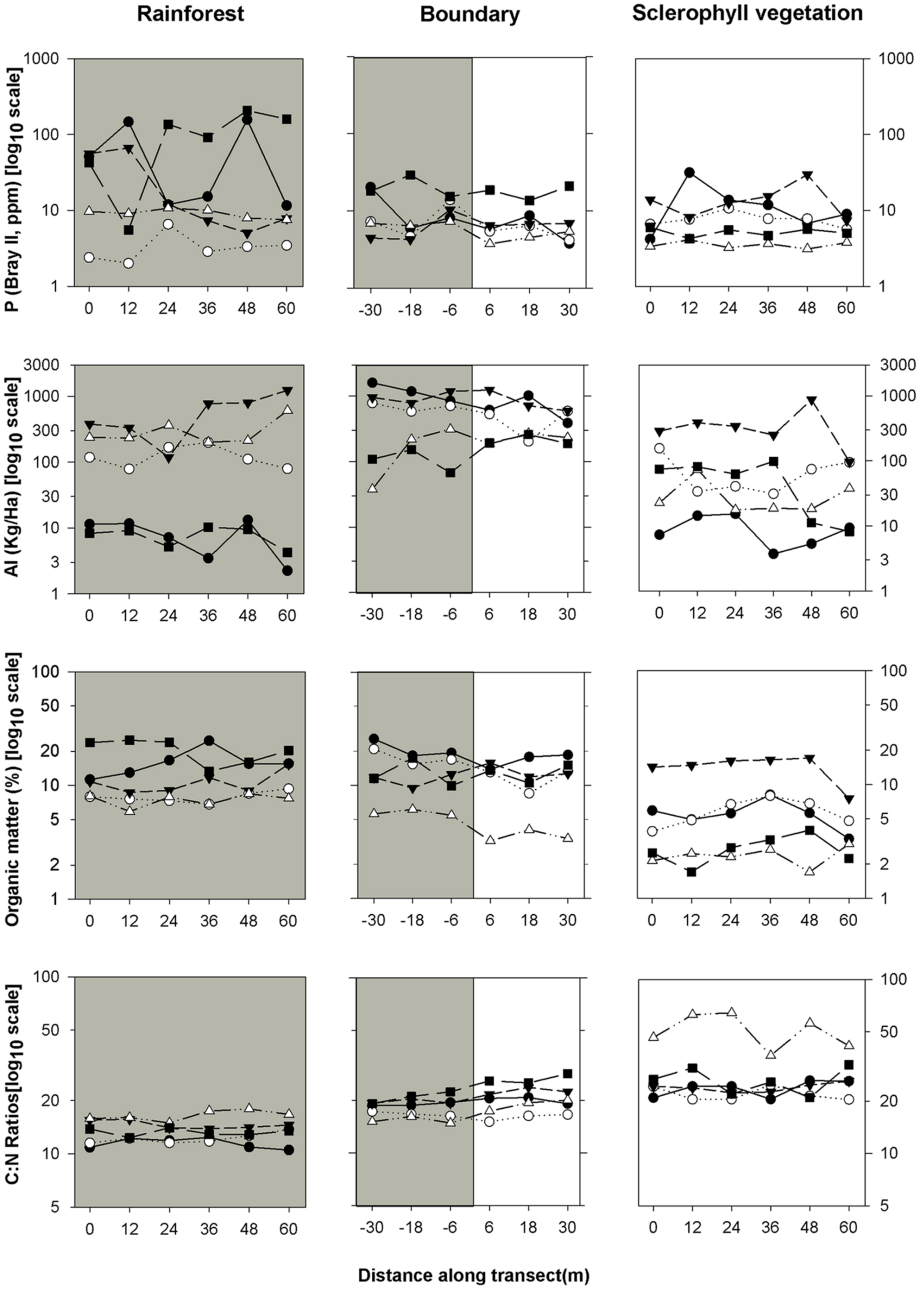
Measurements of soil variables along transects. Measurements of soil variables along transects. Each line represents one transect with six measurements along its length. a) Bray II phosphorus measurements show significantly higher within-transect variance in RF, than across boundaries or SF. b) Aluminium shows high variance in all transects and vegetation types. The Clohesy transect (open triangles) in c) shows the type of pattern expected across boundaries, but overall within-transect variance was not higher across boundaries than within single vegetation types. There was no significant difference in organic matter across boundaries. In contrast, C:N ratios (d) were significantly different across boundaries, but within transect differences were not higher than in RF or SF vegetation. Values for both of these vegetation types were significantly different to the boundary vegetation.

Variance was not significantly higher across boundary transects than in transects wholly within either RF or SF, for any of the 25 soil parameters measured (Figure 3). There were some abrupt changes in parameters across some boundaries (e.g. Figure 3c), but these changes were neither consistent across all parameters or boundaries, nor great enough to result in significantly higher variance for boundary transects overall. The only statistically significant difference in variance was for higher variance in Bray II phosphorus (*p* = 0.045) within a RF transect.

### Vegetation-soil Feedbacks through Time

**Figure 4:**
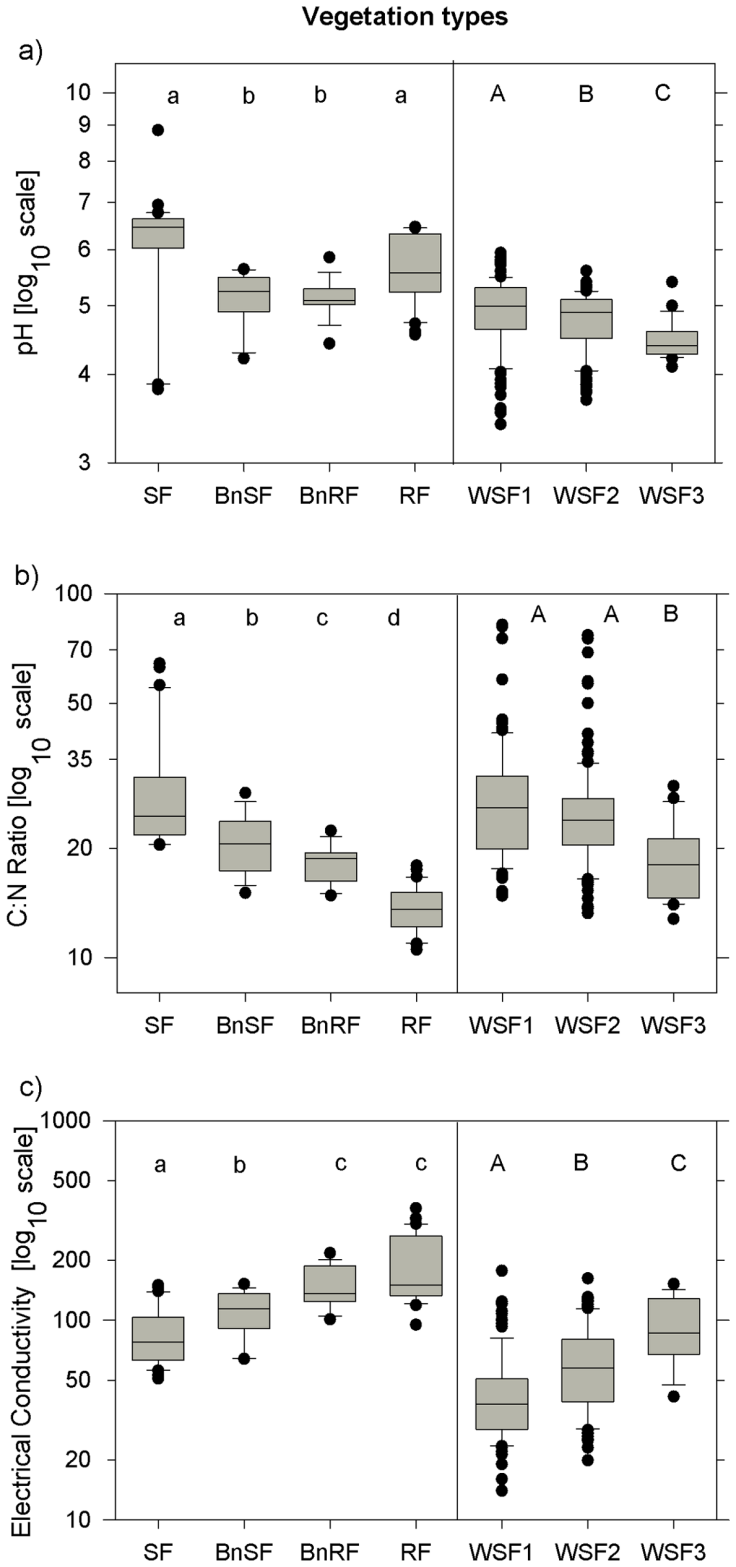
Soil in WSF. Soil parameters measured in sclerophyll vegetation (SF), rain forest (RF), across boundaries in sclerophyll vegetation (BnSF) and rain forest (BnRF), compared to WSF (WSF) with increasing degrees of invasion by rain forest (WSF1, WSF2 and WSF3 respectively). Box plots mark the median and span from the 25^th^ to the 75^th^ percentiles with error bars at the 10^th^ and 90^th^ percentiles. a) C:N values decrease from SF to RF, and with increasing degrees of RF invasion in WSF. A similar but increasing pattern can be seen in b), the electrical conductivity results. In c), pH drops as RF forest invades WSF, even though there is no overall significant difference between RF and SF.

Electrical conductivity, pH, phosphorus and C:N, (but neither carbon nor nitrogen alone) showed significant differences across wet sclerophyll forest with progressive invasion by RF. These parameters, for the most part, followed similar trends to the results we observed from soil under RF and SF. For example, carbon and nitrogen both decreased substantially from RF (means = 7.2% C and 0.55% N), to SF (means = 3.5% C and 0.14% N). Accordingly, C:N ratios dropped sharply from SF (mean = 29.4) to BnSF (mean = 20.8) to RF boundary (mean = 18.3) and again to RF (mean = 13.6), with a significant difference between all vegetation types (*p*<0.005; Figure 4). Likewise, C:N ratios were lower in wet sclerophyll forest with advanced invasion by RF (type III; mean = 18.8, *p* = 0.003) than in either of the two previous stages (which showed no difference between them; means = 28.03 and 25.9, p>0.2).

Values for electrical conductivity (eC; a measure of ions in the soil and a proxy for salinity) in WSF (mean = 57.5 µS cm^−1^) were lower overall than in SF or RF vegetation (means of 86.26 and 191.8 µS cm^−1^ respectively), but they increased with advancing RF invasion (45.32 µS cm^−1^ in Type I forest to 94.41 µS cm^−1^ in Type III) much in the same way as they do from SF to RF (Figure 4, Table 2).

pH dropped significantly with increasing RF invasion (from mean = 4.9 in Type I to means of 4.8 and 4.5 respectively in types II and III; *p*<0.006). The average WSF pH value (4.8) was closer to results from boundary vegetation (mean = 5.1) than either RF (5.66) or SF (6.02). Soil phosphorus decreased (*p*<0.001) from early to mid-stage wet sclerophyll vegetation (Type I to Type II; means = 0.031 and 0.022% respectively), but then showed no difference between mid and late stages (Type II and III; *p* = 0.99) or early and late stages (Type I and III; *p* = 0.49).

## Discussion

### Soil Parameters within and Across Vegetation Types

Contrary to our first hypothesis our results do not, for the most part, indicate a clear set of characteristics that distinguish “boundary zone soil” from contrasting RF and SF soil. They do, however show interesting similarities and contrasts with previous boundary research.

In general, our results showed SF had lower levels of carbon, nitrogen, soil organic matter and the highest C:N ratios, all of which are consistent with higher fire frequencies and the presence of established forests [32], [43], [44]. Higher nitrogen levels, and lower C:N ratios in RF have also been found in previous comparisons of closed and open vegetation, and studies across boundaries in Australia, Africa, India and Brazil [6], [9], [13], [14], [16], [32]. However the cross vegetation-type patterns we found in total nitrogen, and related ammonium and nitrate merit closer consideration as they are indicators of environmental processes other than just fire frequency. Whereas levels of ammonium reflect rates of accumulation of nitrogen through decomposition, nutrient cycling and accumulation of organic matter, lower nitrate levels indicate lower bacterial activity [45]. Our results are therefore consistent with contrasts in both nutrient cycling and bacterial activity across SF-RF boundaries, but varying contrasts and similarities between bacterial activity and nutrient cycling across SF and BNSF, and RF and BnRF. As nitrate is more readily available to vegetation than ammonium, and particularly important to RF pioneer species [46], the relative differences between ammonium and nitrate across vegetation types could play a role in what species and thus which successional pathways are able to establish across boundaries. Mycorrhizae may also play an important role in plant establishment across vegetation types (and in the invasion of WSF by RF), as it is thought they help plants exploit nitrogen in non-nitrate form [46], [47]. Importantly, recent studies have highlighted environmental feedbacks where higher nitrogen availability results in higher phosphorus availability to plants [48]. These feedbacks may be especially important in Australia’s phosphorus limited soils, and explain why so many studies have found links between rainforests and increased phosphorus availability.

It is interesting that two of the phosphorus tests we used (Bray I and II) showed no difference between vegetation types while the third (Colwell) showed values in boundary transects which were intermediate to those in woodland and rain forest (Table 2). Bray measurements are used in agriculture to assess ‘plant available’ phosphorus, while Colwell is quantitative measure of total phosphorus [49]. The applicability of Bray assessments of phosphorus availability for natural plant populations is unclear [4]. Additionally, it has been suggested that Colwell tests may overestimate phosphorus in the soil [50]. Thus, it may be that levels of phosphorus in the soil increase from woodlands to rain forest (as shown by our Colwell test) but not all the phosphorus in rain forests is freely available to plants, especially if it is bound with iron which was also higher in rain forests [50].

Finding significant differences in pH and aluminium between the boundaries and non-boundary vegetation was surprising. Aluminium was the only cation that mirrored pH levels, which may be an indication that soils at the boundaries are acid mineral soils. That is, that exchangeable aluminium cations, rather than hydrogen cations are responsible for the low pH [51]. High aluminium and low pH (<5.5) also indicates potential aluminium toxicity for some species at the boundaries [52]. It is not obvious how aluminium toxicity affects natural communities (in contrast to agricultural vegetation), but it may influence which species can survive in the boundary zone.

By using controls, our results create a context for understanding earlier contradictory studies. Specifically, previous studies in Australian vegetation have found seemingly contradicting results for differences in pH across boundaries. Plowman [31] found pH in WSF to be lower than in RF, while Turton and Sexton [6] found pH to be lower in RF than in SF and attributed the apparent contradiction with the previous study’s [31] results to differences in parent material. We found pH decreased from SF to RF (albeit non-significantly) and was lowest in WSF. Thus, our results are consistent with both studies and suggest that the pH differences described can be attributed to contrasts between ‘wet’ and ‘dry’ sclerophyll vegetation. Using this approach on boundaries elsewhere (such as the cerrado-RF boundaries of Brazil) might allow for better insights into the relative importance of soil chemistry in different systems and for more meaningful comparisons between systems.

Regarding soil physical parameters, our results show RF predominantly on clay soils and most SF on shallower gravelly or sandy soils (Table S1). Although this fits the perceived pattern of RF growing on soils that are better able to retain moisture, Australian RF have not been shown to be restricted to soil with specific textural properties [3]. Indeed, studies throughout the continent have consistently failed to find a relationship between the presence of RF and a variety of physical properties including clay or sand content, field capacity, saturation moisture content, aeration or texture ([3] and references within). None of the boundaries sampled coincided with a change in parent material or soil texture. Similarly, although climatic conditions are sure to have varied between transects, climatic conditions throughout the Australian Wet Tropics are known to be able to support both SF and RF, as well as widespread mosaics of the two vegetation types.

Our results showed that within-transect variance was not higher across boundaries than within-transect variation in either SF or RF vegetation. Together with the results across vegetation types, these results show that considering contrasts across boundaries in the context of non-boundary vegetation and regional soil heterogeneity allows for a more meaningful interpretation of the patterns in soil parameters. Rather than just identifying differences and similarities across boundaries or between vegetation types, this approach gives us a framework to understand whether these differences matter on a larger scale. In this case, our results indicate that contrasts across boundaries alone are less informative than previously thought as non-boundary soils present similar levels of heterogeneity to those at the boundary. Using appropriate non-boundary controls could provide important context and insights in studies of edge-effects, tree lines, and a wide variety of comparisons between adjacent contrasting communities and ecosystems.

Our study shows interesting contrasts and similarities with savanna-forest boundaries in Brazil and India. For example, a series of studies [9], [13], [16] have found differences across boundaries in parameters including carbon, magnesium and iron, where we did not. However at a larger scale, we did find differences in these elements between SF and RF. These differences and similarities may be due to stronger chemical contrasts across boundaries outside the Australian Wet Tropics, but the previous results might become more similar to ours if comparisons to non-boundary vegetation and of variance along boundaries were incorporated. It may also be that grassland-forest boundaries present stronger chemical contrasts than do woodland-closed forest boundaries because of more similar litter-cycling in the latter [7].

### Changes in Soil through Time

The results from our first two questions become particularly interesting in light of the results from the third. The accumulation of certain nutrients (particularly nitrogen and carbon) by RFs has been discussed previously both in Australian and global contexts (e.g. [19], [32]). Our data support the hypothesis that as RF invades WSF, RF vegetation itself changes the soil. With increased RF invasion, over periods of a few hundred years, the soil under wet sclerophyll vegetation becomes increasingly similar to RF soil. Taking vegetation-soil feedbacks into account allows for a different, more dynamic interpretation both of soils and vegetation at the boundaries, and is of particular interest to studies related to ASS. For example, soil-vegetation feedbacks may help explain why both sides of the boundaries tend to be more similar to RF, and why we found no cases where soil parameters in RF were different to the other vegetation types while this was often the case for SFs. If the boundaries between RF and SFs are relatively fluid and have continued to move back and forth across the landscape through time, as would be expected following ASS theory [23] then we should expect to find the “signature” of recent RF inputs into the soil near current boundaries.

An unexpected outcome of our study was finding that several important parameters in wet sclerophyll forest soils were, on average, more similar to boundary transects than to either RF or SF soils. This raises interesting questions relating to differences between transitional soils in comparison to those of established vegetation (and perhaps transitional vegetation states, sensu [23]). A series of environmental processes related to species changes and changing site conditions (e.g. shade, soil moisture maxima and minima) could result in differences between soil deep within tracts of established vegetation and soils at the boundaries and in transitional zones like the wet sclerophyll forests. The role of species from different successional stages feeding back different elements into the soil could be particularly interesting in this context. For example, high levels of aluminium could be linked to species that are hyper-accumulators (such as *Ceratopetalum apetalum*) [53]. Species of *Gossia* and *Macadamia*, both RF genera found in far north Queensland have recently been found to accumulate metals in their foliage (including manganese, which presented particularly complex patterns of contrast and similarities across vegetation types, Figure 2) [54].

The wet sclerophyll forests of north-eastern Australia provide a window into the processes of transition from open to closed vegetation, and these forests seem to have similarities with the wet savannas and cerradão of South America [55]. Comparing boundary to non-boundary vegetation in these South American vegetation types, and elsewhere in the world may provide interesting insights and comparisons into the dynamics of boundaries and transitions between open and closed forests. Obtaining a better understanding of abrupt vegetation boundaries and the transitions between vegetation types could prove to be especially important in understanding large-scale and long-term vegetation dynamics, especially in the face of climate change and for restoration and conservation worldwide.

### Conclusions

Our first finding was that soil parameters across boundaries did not correspond entirely either to “boundary soil” or contrasting “RF soil and SF soil”. Rather, our results reflect a complex mix of feedbacks, vegetation ‘footprints’ and ongoing processes at a variety of scales. Our second main finding was that variance in soil parameters was not higher across boundaries than within vegetation types. Thus, to be able to interpret and understand processes occurring across boundaries, it is important to include controls in both vegetation types, away from the boundaries. Our findings have strong implications for other boundary and edge effect studies in ecology, not just in tropical vegetation, but as far reaching as studies of boundaries caused by currents and eddies in marine systems (e.g. [56]). Our third main finding was that RF vegetation transforms “wet sclerophyll forest soil” into “rain forest soil”. Taken together, our results present a comprehensive picture of plant-soil feedbacks in both boundary and non-boundary vegetation in Australia’s Wet Tropics. Our results have implications for studies comparing transitional and established vegetation, studies of ASS, and possibly for restoration efforts as well. Perhaps more importantly, our study emphasizes the importance of leaving behind the pervasive view of RFs as static, pyrophobic and monolithic and incorporating more dynamic long-term views of landscape processes and interactions between vegetation types and communities.

## Supporting Information

Figure S1 **Characteristic boundary between closed rainforest and woodland dominated by grasses, cycads and eucalypts.**
(TIF)

Figure S2 **Woodland near the Davies Creek Site.**
(TIF)

Figure S3 **Complex rainforest in the Daintree.**
(TIF)

Figure S4 **Wet sclerophyll forest.** ***Eucalyptus grandis*** **over a grassy understorey.**
(TIF)

Table S1 **Site descriptions.**
(DOC)
