# Supplementary figures and images for: A Broad Approach to Abrupt Boundaries: Looking Beyond the Boundary at Soil Attributes within and Across Tropical Vegetation Types
Source: PLoS One. 2013 Apr 10;8(4):e60789. doi: 10.1371/journal.pone.0060789 (PMC3622688; doi:10.1371/journal.pone.0060789)

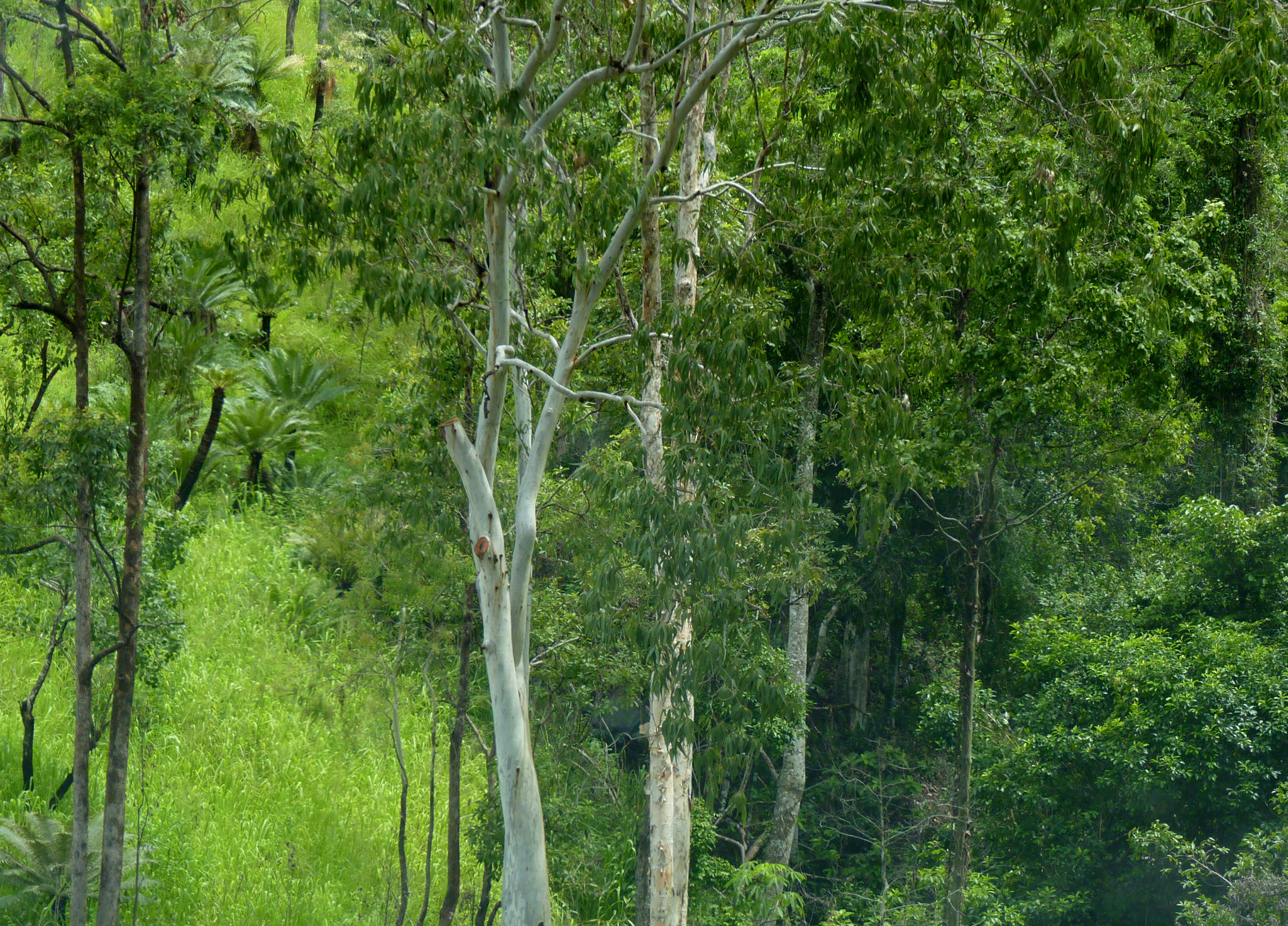

Supplement: Figure S1 — Characteristic boundary between closed rainforest and woodland dominated by grasses, cycads and eucalypts. (TIF) [file pone.0060789.s001.tif]

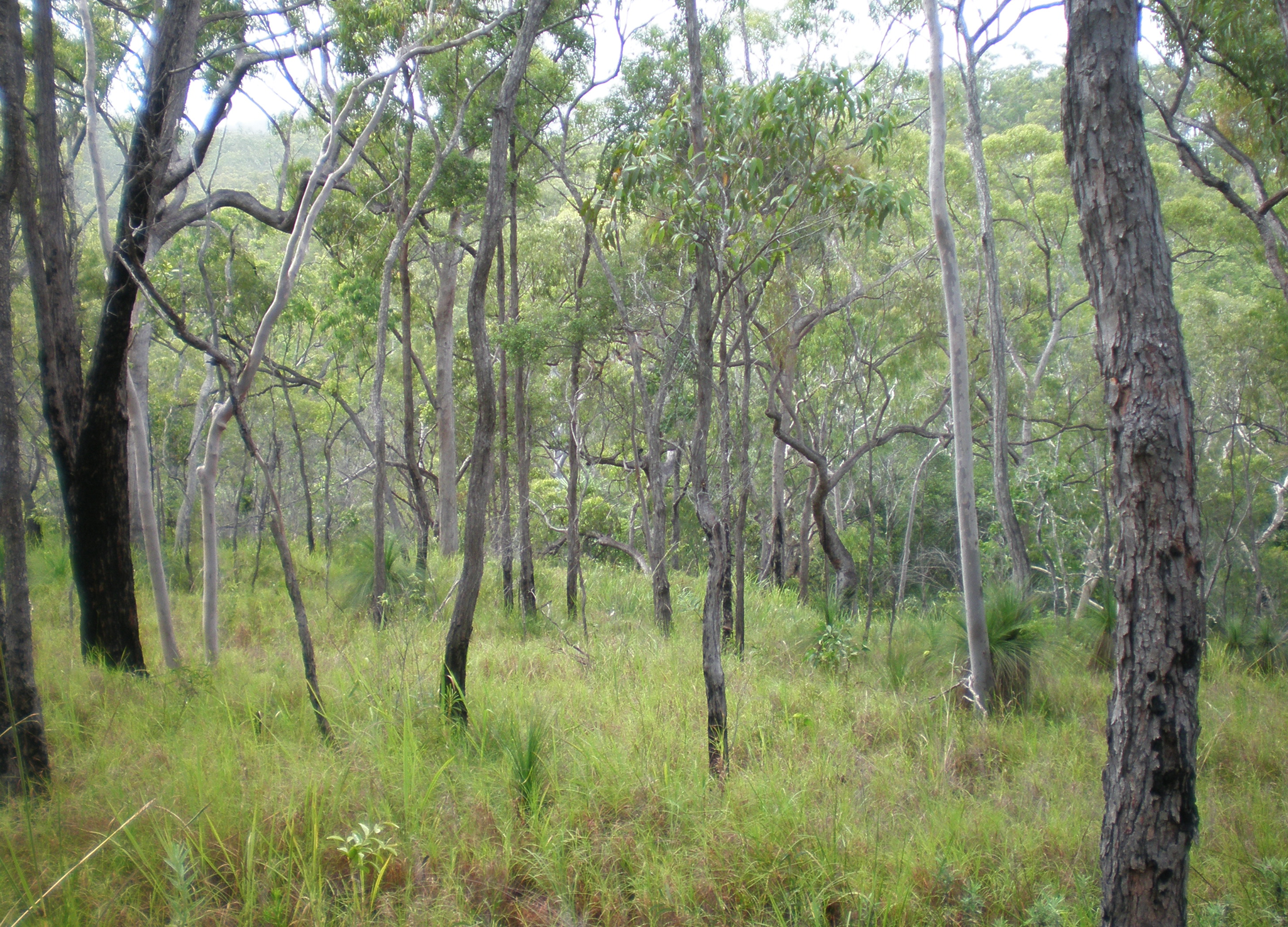

Supplement: Figure S2 — Woodland near the Davies Creek Site. (TIF) [file pone.0060789.s002.tif]

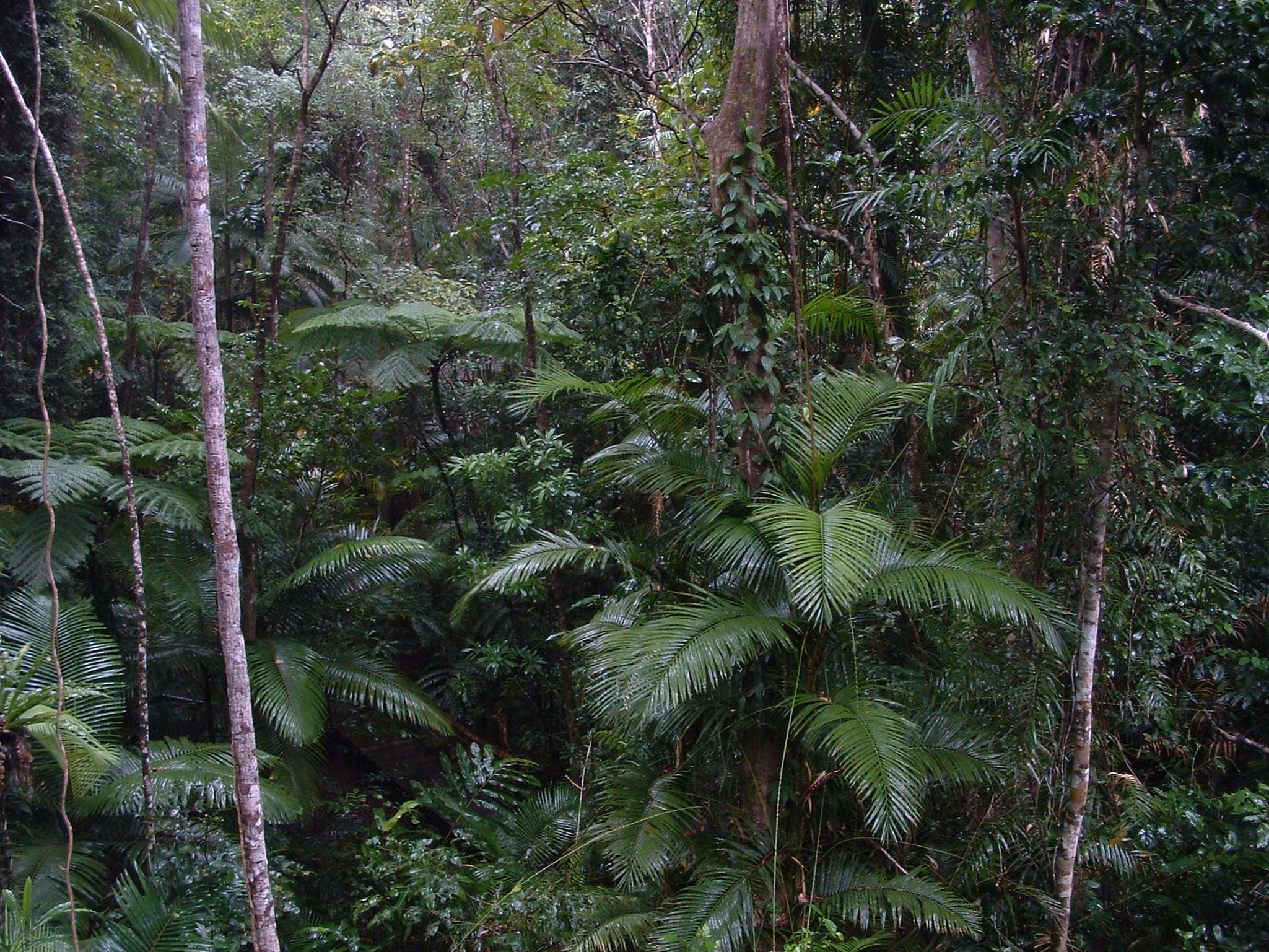

Supplement: Figure S3 — Complex rainforest in the Daintree. (TIF) [file pone.0060789.s003.tif]

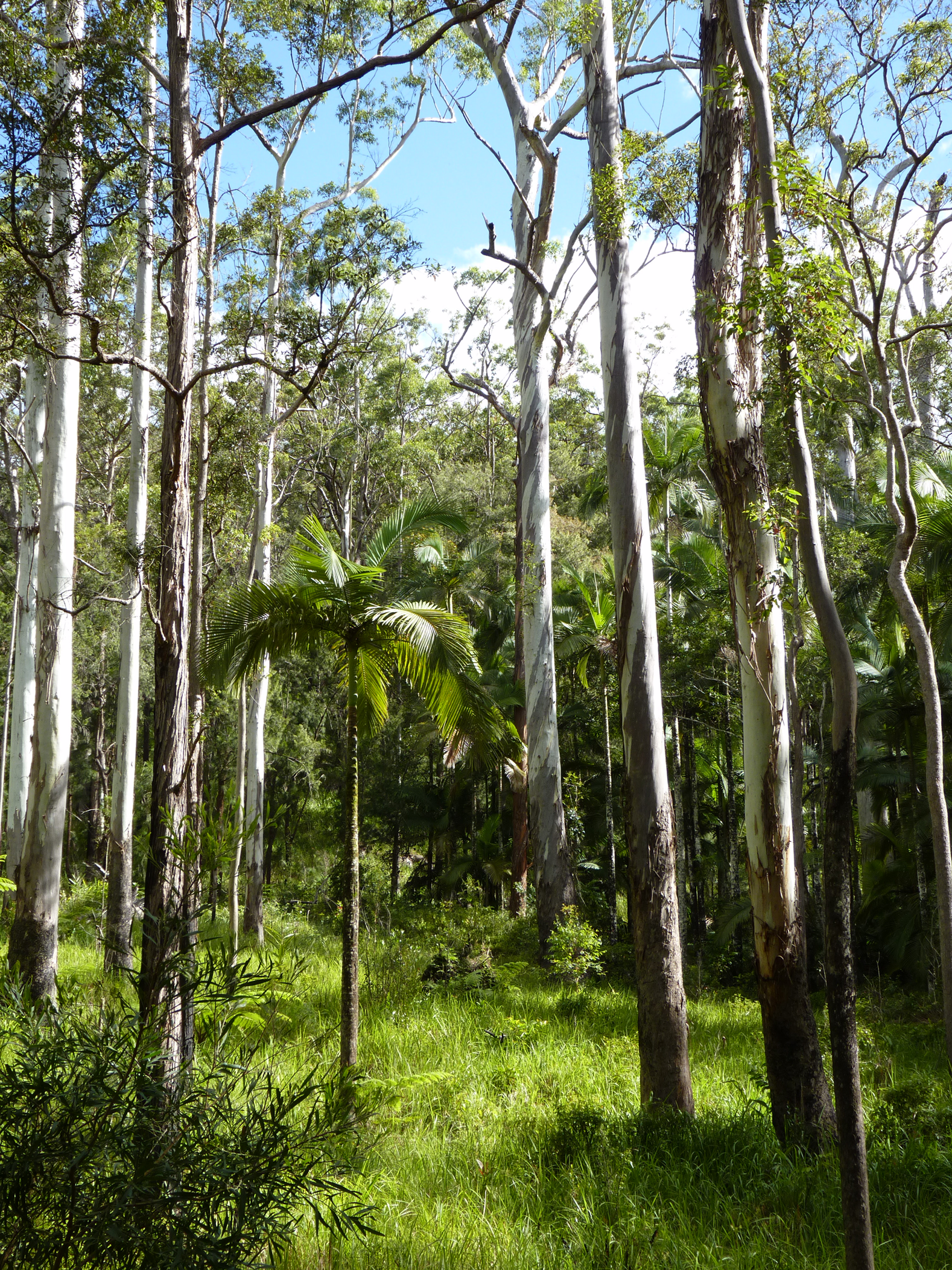

Supplement: Figure S4 — Wet sclerophyll forest. Eucalyptus grandis over a grassy understorey. (TIF) [file pone.0060789.s004.tif]
