# Supplementary material for: A Broad Approach to Abrupt Boundaries: Looking Beyond the Boundary at Soil Attributes within and Across Tropical Vegetation Types
Source: PLoS One. 2013 Apr 10;8(4):e60789. doi: 10.1371/journal.pone.0060789 (PMC3622688; doi:10.1371/journal.pone.0060789)
Supplement: Table S1 — Site descriptions. (DOC) [file pone.0060789.s005.doc]

**Table S1 - Site descriptions**

| Site | Altitude (m) | Location | Description |
| --- | --- | --- | --- |
| Surprise Creek | 383 | 16° 51.328' S, 145° 38.435' E | Sclerophyll woodland with grass and sedge understorey, occasional mossy beds and terrestrial orchids near a treefall. Metamorphic, whitish soils. |
| Davies Creek (2) | 456 | 16° 59.885' S, 145°33.897' E | Woodland site, with dense understorey of sedges and grasses and regenerating woody plants under sparse *Eucalyptus* spp. Red clay soil. |
| Davies Creek (1) | 623 | 17°00.714' S, 145°34.960' E | Sclerophyll vegetation. Grassy (*Themeda spp*.) understorey with *Xanthorrhea* *sp*. *Acacia spp.* and *Allocasuarina* *torulosa* in the canopy. Greyish gravelly soil, bare granitic rock in spots. |
| Robson Track | 280 | 17° 9.356' S, 145°42.088' E | Woodland with *Allocasuarina torulosa*, *Eucalyptus* spp., *Acacia* spp. and a *Themeda triandra* understorey. Shallow sandy soils over granite. |
| Mt. Baldy | 847 | 17° 16.704' S, 145° 27.765' E | Woodland with open *Themeda triandra* understorey and some cycads. *Eucalyptus* and *Allocasuarina* *torulosa* canopy. Shallow soils over rhyolite. |
| Jindalba | 353 | 16° 14.413' S, 145° 25.956' E | Lowland rain forest within Daintree National Park. Complex mesophyll vine forest. Canopy to 30-40 m. Trees with large buttresses, many vines and epiphytes. Thick reddish clay. |
| Smith's Track | 449 | 16°53.327' S, 145°38.813' E | Rain forest with open understorey under a dense canopy. Plentiful *Calamus* spp. and *Alyxia ruscifolia*; mature *Agathis robusta,* *Podocarpus sp*. and *Gossia* spp. present. Soil with high clay content. |
| Eacham | 756 | 17° 17.216' S, 145° 37.776' E | Mature upland rain forest on basalt. Relatively dense understorey. *Ficus spp.*, *Argyrodendron* spp. and *Gossia* spp.common. Dense orange soil with high clay content. |
| Hypipamee | 789 | 17° 25.805' S, 145° 29.201' E | Rain forest with remarkably sparse and open understorey under a dense and tall canopy. *Psychotria nematopoda*, *Ficus* spp*.*, *Calamus* spp*.* present. |
| Nandroya | 354 | 17° 35.407' S, 145° 45.270' E | Mature lowland rain forest with a canopy to 30 m. Samples taken near buttresses of large *Ficus* spp. Shallow soil with clay, numerous roots in the soil. Basaltic parent material. |
| Surprise (Boundary) | 410 | 16° 51.567' S, 145° 39.018' E | Dense rain forest with many ferns in sparse understorey. *Ficus* spp., *Agathis robusta* and *Calamus* spp*.* in canopy. Sedges plentiful in woodland understorey, under *Acacia* spp. and, *Syncarpia glomulifera*. Fine basaltic black soil. |
| Smith's Track (Boundary) | 382 | 16° 53.061' S, 145° 39.059' E | Rain forest canopy to 25-30 m, shrubby understorey. Very deep leaf litter in woodland, abundant grasses. *Eucalyptus* spp. and *Allocasuarina torulosa* with strongly scorched trunks. |
| Clohesy  (Boundary) | 407 | 16° 55.630' S, 145° 36.257' E | Abundant grasses in woodland, under *Corymbia torreliana* and *Eucalyptus tereticornis*. Rain forest understorey very open in comparison, but very strongly shaded with no grass. |
| Herberton (Boundary) | 760 | 17° 16.428' S, 145° 25.774' E | Open forest with dense grassy understorey under a few eucalypts abutting dense rain forest with a relatively open understorey. Plagued by leeches from the very bowels of hell. |
| Hypipamee (Boundary) | 789 | 17° 25.691' S, 145° 29.178' E | Boundary between young rain forest with a canopy to 20m and open forest with dense understorey of sedges and grasses. Shallow soils over granite. |

Note that coordinates are often approximate, taken from the nearest site where it was possible to obtain a GPS signal.
